# Indoxyl sulfate induced frailty in patients with end-stage renal disease by disrupting the PGC-1α–FNDC5 axis

**DOI:** 10.18632/aging.205141

**Published:** 2023-10-23

**Authors:** Yi-Chou Hou, Min-Tser Liao, Kuo-Wang Tsai, Cai-Mei Zheng, Hui-Wen Chiu, Kuo-Cheng Lu

**Affiliations:** 1 Division of Nephrology, Department of Internal Medicine, Cardinal Tien Hospital, New Taipei City 231, Taiwan; 2 School of Medicine, Fu Jen Catholic University, New Taipei City 242, Taiwan; 3 Department of Pediatrics, Taoyuan Armed Forces General Hospital, Taoyuan 325, Taiwan; 4 Department of Pediatrics, Tri-Service General Hospital, National Defense Medical Center, Taipei 114, Taiwan; 5 Department of Medical Research, Taipei Tzu Chi Hospital, Buddhist Tzu Chi Medical Foundation, New Taipei City 231, Taiwan; 6 Department of Internal Medicine, Division of Nephrology, Shuang Ho Hospital, School of Medicine, College of Medicine, Taipei Medical University, New Taipei City 110, Taiwan; 7 TMU Research Centre of Urology and Kidney, Taipei Medical University, New Taipei City 110, Taiwan; 8 Graduate Institute of Clinical Medicine, College of Medicine, Taipei Medical University, New Taipei City 110, Taiwan; 9 Department of Medical Research, Shuang Ho Hospital, Taipei Medical University, New Taipei City 110, Taiwan; 10 Division of Nephrology, Department of Medicine, Taipei Tzu Chi Hospital, Buddhist Tzu Chi Medical Foundation, New Taipei City 231, Taiwan; 11 Division of Nephrology, Department of Medicine, Fu Jen Catholic University Hospital, School of Medicine, Fu Jen Catholic University, New Taipei City 243, Taiwan

**Keywords:** indoxyl sulfate, sarcopenia, frailty, irisin, PPARγ, PGC-1α, FNDC5, resveratrol

## Abstract

Objective: Sarcopenia or frailty is common among patients with chronic kidney disease (CKD). The protein-bound uremic toxin indoxyl sulfate (IS) is associated with frailty. IS induces apoptosis and disruption of mitochondrial activity in skeletal muscle. However, the association of IS with anabolic myokines such as irisin in patients with CKD or end-stage renal disease (ESRD) is unclear. This study aims to elucidate whether IS induces frailty by dysregulating irisin in patients with CKD.

Materials and Methods: The handgrip strength of 53 patients, including 28 patients with ESRD, was examined. Serum concentrations of IS and irisin were analyzed. CKD was established in BALB/c mice through 5/6 nephrectomy. Pathologic analysis of skeletal muscle was assessed through haematoxylin and eosin and Masson’s trichrome staining. Expression of peroxisome proliferator-activated receptor-gamma coactivator PGC-1α and irisin were analyzed using real-time polymerase chain reaction and Western blotting.

Results: Handgrip strength was lower among patients with ESRD than among those without ESRD. In total, 64.3% and 24% of the patients in the ESRD and control groups had low handgrip strength, respectively (*p* < 0.05). Serum concentrations of IS were significantly higher in the ESRD group than in the control group (222.81 ± 90.67 μM and 23.19 ± 33.28 μM, respectively, *p* < 0.05). Concentrations of irisin were lower in the ESRD group than in the control group (64.62 ± 32.64 pg/mL vs. 99.77 ± 93.29 pg/mL, respectively, *p* < 0.05). ROC curves for low handgrip strength by irisin and IS were 0.298 (95% confidence interval (CI): 0.139–0.457, p < 0.05) and 0.733 (95% CI: 0.575–0.890, p < 0.05), respectively. The percentage of collagen was significantly higher in mice with 5/6 nephrectomy than in the control group. After resveratrol (RSV) treatment, the percentage of collagen significantly decreased. RSV modulates TGF-β signaling. In vitro analysis revealed that IS treatment suppressed expression of PGC-1α and FNDC5 in a dose–dependent manner, whereas RSV treatment attenuated IS-induced phenomena in C2C12 cells.

Conclusion: IS was positively correlated with frailty in patients with ESRD through the modulation of the PGC-1α–FNDC5 axis. RSV may be a potential drug for reversing IS-induced suppression of the PGC-1α–FNDC5 axis in skeletal muscle.

## INTRODUCTION

Chronic kidney disease (CKD) is defined as the chronic decline in the glomerular filtration rate or structural abnormality of the genital urinary tract [1]. The incidence of CKD has increased over time along with global awareness of the disorder. The development of comorbidities among patients with CKD during the aging process, such as diabetes mellitus, primary hypertension, congestive heart failure, and chronic glomerulosclerosis, has also increased [2, 3]. Uremic toxins, which are hazardous metabolites, are excreted at a decreased rate in patients with CKD due to impaired functioning of the glomerulus and proximal tubules. Indoxyl sulfate (IS) is classified as a protein-bound uremic toxin [4]. It has been associated with the pathogenesis of CKD-related comorbidities such as vascular intimal hyperplasia, tubulointerstitial fibrosis, left ventricular remodeling, renal osteodystrophy, and sarcopenia [5, 6].

Frailty is a natural part of the aging process and is characterized as a progressive decline of physical function. Sarcopenia, a subset of frailty, is defined as the loss of skeletal muscle both in terms of quality and quantity [7]. A decrease in skeletal muscle and fatigue increases the risk of negative health-related events such as hospitalization or falls [8]. The factors contributing to sarcopenia are multifactorial and include altered immunosenescence, decreased insulin resistance, chronic inflammation, oxidative stress, decreased caloric intake, decreased number of satellite cells, microvascular injury, and inactivity or disuse due to systemic illness [9, 10]. To counteract sarcopenia, stimulation of anabolic pathways is essential. An axis of three anabolic pathways that is crucial in the anabolism of skeletal muscle involves peroxisome proliferator–activated receptor gamma (PPARγ), PPARγ coactivator 1-alpha (PGC-1α), and fibronectin type III domain containing 5 (FNDC5) [11, 12]. Under conditions of oxidative stress, PGC-1α and PPARγ modulate levels of exercise-related anabolic myokines such as irisin [12]. Irisin has been shown to reduce white adipose tissue by increasing thermogenesis and stimulating brain-derived neurotrophic factors. This could potentially preserve cognitive and physical function [13, 14]. Frailty is more common among patients with CKD than among those without CKD, and altered PGC-1α-FNDC5 (irisin) expression within skeletal muscles has been proposed as a possible pathogenesis of frailty.

IS is a protein-bound uremic toxin that is metabolized from indole, a compound that is generated by the degradation of tryptophan by gut microbiota. IS is excreted from the renal tubules by organic anion transporters. IS causes intracellular oxidative stress and influences transcription via the aryl hydrocarbon receptor [5], resulting in increased apoptosis. Furthermore, disruption of the differentiation of particular progenitor cells or mesenchymal stem cells can impede the body’s ability to repair various organ systems [15]. Decreasing protein-bound uremic toxins is a therapeutic strategy for reducing the risk of organ damage among patients with CKD. This study investigates whether IS, by modulating the PGC-1α–FNDC5 axis, causes skeletal muscle dysfunction and whether resveratrol (RSV) attenuates the effects of IS.

## MATERIALS AND METHODS

### Ethics and participant recruitment

This study was performed at a regional hospital in New Taipei City, Taiwan, from August 2018 to January 2020 under the tenets of the Declaration of Helsinki. The study was approved by the Ethics Committee of Human Studies at Cardinal Tien Hospital (CTH-107-3-5-027). Patients were included if they had an estimated GFR (eGFR) of <60 mL/min or spot urine proteinuria of >200 mg/g and were aged >20 years and able to communicate verbally in Mandarin Chinese. Patients were excluded if they had unstable angina, acute myocardial infarction within the past 6 months, severe anemia (Hb < 8 g/dL), systolic hypertension (>190 mmHg), active inflammation or infection, cancer, autoimmune diseases, emotional instability, musculoskeletal disability, uncontrolled cardiac failure, or respiratory problems or were hospitalized within the past month. Patients were divided into three groups: (1) the control group (eGFR > 60 mL/min), (2) the CKD group (eGFR 15–60 mL/min), and (3) the end-stage renal disease (ESRD) group. ESRD was defined as receiving maintenance renal replacement therapy (hemodialysis or peritoneal dialysis) continuously for >3 months. Patients receiving hemodialysis received conventional hemodialysis with a high-flux or high-efficiency dialyzer.

### Measurement of myokines and indoxyl sulfate

Myokines (irisin, myostatin, and interleukin-6) were measured using an enzyme immunoassay kit (Abbkine, Wuhan, China). Serum samples were drawn to measure biochemical and hematological parameters. The serum was collected under fasting conditions and stored at −80°C for later measurement. Parameters were measured according to the manufacturer’s instructions (the inter- and intra-assay coefficients of variability for irisin, myostatin, and interleukin-6 were <11% and <9%, respectively). IS was measured using an enzyme-linked immunosorbent assay (ELISA) kit (Leadgene Biomedical, Tainan, Taiwan) and validated through high-performance liquid chromatography–mass spectrometry (Leadgene, Tainan City, Taiwan. US patent: US10723791B2). The serum was diluted 20-fold to a final volume of 100 μL and then added to an equal volume of diluted detection antibody. After 1 h, the ELISA wells were washed, and 3,3′,5,5′-tetramethylbenzidine was used for color development. IS levels were determined based on a standard curve.

### Handgrip strength

A hand dynamometer (Camry Electronic Co. Ltd., Guangdong, China) was used to measure grip strength. Patients were asked to use the dynamometer in a standing position with the forearm stretched away from the body at thigh level. Patients applied the maximum grip strength of the dominant hand. For patients receiving hemodialysis, handgrip strength was measured by using the hand without an arteriovenous fistula. Three measurements for each patient were obtained with resting intervals of at least 30 s. Low handgrip strength was defined as <28 kg in men and <18 kg in women.

### Cell line

C2C12 cells, an immortalized myoblast cell line from mice, were obtained from the American Type Culture Collection. The cells were cultured in Dulbecco’s modified Eagle’s medium (DMEM; Thermo Fisher Scientific, Boston, MA, USA) containing 10% fetal bovine serum (Gibco, Thermo Fisher Scientific, Waltham, MA, USA) and 1% penicillin–streptomycin (FUJIFILM Wako Pure Chemical, Richmond, VA, USA) in a humid environment containing 5% CO2 at 37°C. The cells were seeded at a density of 7.5 × 10^5^ per 35-mm dish for 24 h. To promote differentiation, the cells were cultured for another 24 h in DMEM containing 2% horse serum (Thermo Fisher Scientific) after reaching 90–100% confluence. The cells were subsequently treated with different doses (0, 125, 250, and 500 μM) of IS (IS potassium salt; Sigma-Aldrich, St. Louis, MO, USA) for 24 and 48 h. To evaluate the effect of RSV (Tokyo Kasei Kogyo Co., Tokyo, Japan), 10 μM of RSV was added to the DMEM containing 2% horse serum and a batch of cells was cultured in this medium for 3 h prior to being treated with IS.

### Western blot assay

Muscle tissue cells were homogenized in radioimmunoprecipitation assay buffer (50 mM Tris at pH 7.4, 150 mM NaCl, 1% Triton X-100, 1% SDS, 1% deoxycholate with complete protease inhibitor cocktail; Roche, Penzberg, Germany). Lysates were collected and centrifuged to remove cell debris. Protein concentration was evaluated by using the Bio-Rad DC Protein Assay Kit (500-0113, Bio-Rad Laboratories, Inc., Hercules, CA, USA), which is based on the Bradford dye-binding procedure. Protein samples (60 μg) were separated through SDS-PAGE in 10% resolving gel by using a Mini-PROTEAN 3 Cell apparatus and then electrotransferred to nitrocellulose membranes (Amersham Pharmacia Biotech, Amersham, UK). The membranes were blocked with 1% skimmed milk in Tris-buffered saline–Tween overnight and then incubated with primary antibodies PGC-1α (Proteintech Group, Inc., Rosemont, IL, USA; 66369-1, 1:1000), FNDC5 (Abcam, Cambridge, UK, No: ab131390, 1:1000), myoD (Santa Cruz Biotechnology, Santa Cruz, CA, USA, No: sc-377460; 1:100), and β-actin (Merck, Darmstadt, Germany, No: MAB1501, 1:1000) overnight at 4°C. The membranes were subsequently incubated with antirabbit or antimouse IgG HRP-conjugated secondary antibodies (1:10000, Roche Molecular Biochemicals, Basel, Switzerland) for 1 h at room temperature. After three washes with PBS-Tween, immunoreactive bands were detected using WesternBright ECL (K-12045-D50, Advansta, Inc., Menlo Park, CA, USA).

### SMAD luciferase reporter assays

To assess the effect of mechanism of extracellular matrix fibrosis, the Smad-binding element luciferase reporter lentivirus was applied. SMAD binding element (SBE) luciferase reporter lentiviral particles infected the C2C12 cells in white opaque 96-well plates. After 48 hours, the luminescence assay was performed by the One-Step Luciferase Assay System (BPS Biosciences, San Diego, CA, USA) according to the manufacturer’s instructions. After luciferase assay transfected, C2C12 cells were treated with indoxyl sulfate by 250 uM. To assess the effect of resveratrol for indoxyl sulfate, C2C12 cells were treated with resveratrol at 10 uM.

### Mouse study

The animal study was approved by the Institutional Animal Care and Use Committee of Taipei Tzu-Chi Hospital (IACUC-110-001, approved on February 2021) following the National Institutes of Health Guidelines. Male BALB/c mice (aged 8 weeks and with a body weight of more than 20 g) were obtained from BioLASCO Taiwan Co., Ltd (Taipei, Taiwan) and divided into treatment and control groups. The mice in the treatment group underwent 2/3 nephrectomy of the right kidney followed one week later through removal of the whole left kidney. The mice in the control group underwent sham surgeries (back incisions) on each side. After 12 weeks, the mice received daily intraperitoneal injections for 4 weeks with normal saline (110 λ/mouse) or RSV (30 mg/kg/day, approximately 110 λ/mouse). The goal of administering RSV was to reduce the IS burden.

The mice were divided into four groups: sham + normal saline (NS, *n* = 10), sham + RSV (*n* = 10), 5/6 nephrectomy + NS (*n* = 10), and 5/6 nephrectomy + RSV (*n* = 10) [16]. Each group had six mice and totally 40 mice. All mice were kept in pathogen-free animal facilities under controlled conditions at 22°C with a 12-h light/dark cycle. The mice were sacrificed after 12 weeks and their gastrocnemii were analyzed.

### Histological analysis of gastrocnemius

The gastrocnemii were fixed in 10% buffer formalin and then embedded in paraffin. Hematoxylin and eosin (H&E) staining and Masson’s trichrome staining (Muto Pure Chemical Co., Tokyo, Japan) were performed according to the standard protocols on 5-μm–long sections of deparaffinized gastrocnemii.

In Masson’s trichrome staining, collagen fibers were stained with aniline blue. The slides were visualized with an IX73 microscope (Olympus, Tokyo, Japan), a Q imaging Retina 3000 camera, and Q capture Pro7 software. The nucleus of skeletal muscle and the collagen were counted in 10 fields of the same slice at 10× magnification. The nuclei were counted with H&E staining. The number of nuclei was determined according to the average number of nuclei in each image field. In Masson’s trichome staining, we measured the area of collagen (in the blue stain) in each image in ImageJ software. The area of collagen was determined according to the average area of blue stain per image. The numbers of interstitial nuclei and muscle nuclei and the area of interstitial space were quantified in ImageJ software. All histological data were presented as average results from three random, nonoverlapping image fields captured under a 20× objective [17].

### RNA extraction and real-time PCR

Total RNA was isolated using a TRIzol reagent (Invitrogen; Thermo Fisher Scientific Inc., Waltham, MA, USA) according to the manufacturer’s instructions. RNA was quantified using a Nanodrop 2000 device (Thermo Fisher Scientific). In total, 2 μg of total RNA was reverse transcribed with oligo (dT) 15 primers and SuperScript III Reverse Transcriptase according to the manufacturer’s instructions (Invitrogen; Thermo Fisher Scientific). Real-time quantitative polymerase chain reaction (qPCR) was conducted using the ABI stepOneTM system (Applied Biosystems; Thermo Fisher Scientific Inc., Waltham, MA, USA) to measure the expression of specific genes. Individual gene expression was performed with gene-specific primers, and gene expression was detected using a SYBR Green I assay (Applied Biosystems; Thermo Fisher Scientific). The reverse transcription polymerase chain reaction primers are listed below. All primers were synthesized by Genomics (Taipei, Taiwan). mRNA expression of GAPDH was used as an internal control to normalize gene expression in each sample.

**Table:** 

	**Forward**	**Reverse**
*Irisin*	GCTAGGCTGCGTCTGCTTC	AGCCAATGACCACTTCATCC
*TGF-βr1*	CCTCGAGACAGGCCATTTGT	AGACGAAGCAGACTGGACCA
*TGFβr2*	ACGAGCCCCCATTTGGTT	CTCAGCACACTGTCTTTCATGCT
*TGFβ3*	GTGTACGCCCCCTTTATATTGACT	GGTTCGTGGACCCATTTCC
*GAPDH*	GCATCTTCTTGTGCAGTGCC	ACTGTGCCGTTGAATTTGCC

### Statistics

Continuous data are expressed as mean ± standard deviation. Categorical data are expressed in percentages. One-way analysis of variance was used to compare the differences in variables within the three patient groups. Receiver operating characteristic (ROC) curves were plotted, and the area under the curve was estimated. All statistical analyses were performed using the statistical package SPSS for Windows (v.17, SPSS, Chicago, IL, USA). A two-tailed *p* value of < 0.05 was considered statistically significant.

## RESULTS

### Indoxyl sulfate was associated with lower handgrip strength with counteracting the effect of irisin in patients with CKD

**Figure 1:**
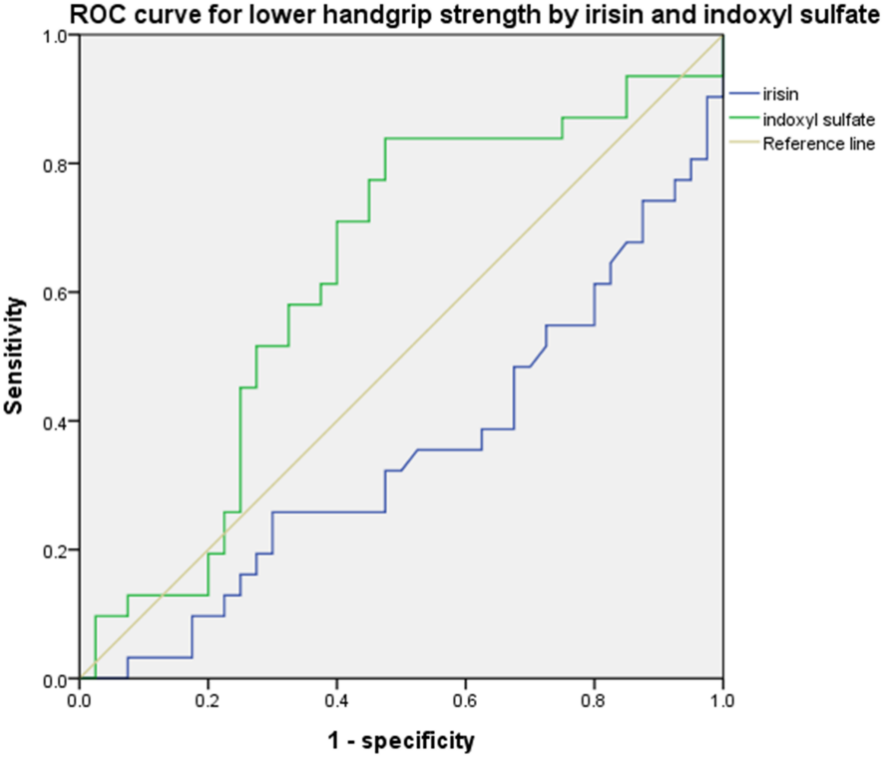
**ROC curve for handgrip strength.** Irisin: 0.298 (95% CI: 0.139–0.457) and indoxyl sulfate: 0.733 (95% CI: 0.575–0.890).

The number of patients with low handgrip strength is detailed in Table 1. The mean handgrip strength was 30.38 ± 9.53 kg in the control group and 21.87 ± 10.04 kg in the ESRD group. The proportion of patients with low handgrip strength was higher in the ESRD group than in the control group (64.3% vs. 24%, respectively, *p* < 0.05). Clinical parameters are shown in Table 2. The concentrations of IS were 222.81 ± 90.67 and 23.19 ± 33.28 μM in the ESRD and control groups, respectively (*p* < 0.05). The concentrations of irisin were higher in the control group than in the ESRD group (99.77 ± 93.29 pg/mL vs. 64.62 ± 32.64 pg/mL, respectively, *p* < 0.05). Figure 1 shows the ROC curves of IS and irisin for predicting low handgrip strength. The ROC curves for irisin and IS were 0.298 (95% confidence interval (CI): 0.139–0.457) and 0.733 (95% CI: 0.575–0.890), respectively.

**Table 1:** Comparison of handgrip strength between patients with and without ESRD.

	**Control group**	**ESRD**	***P* value**
Sample size	25	28	
Age	56.84 ± 10.30	58.85 ± 9.73	*P* = 0.467
Grasping power (kg)	30.38 ± 9.53	21.87 ± 10.04	*P* < 0.05
Case number of lower handgrip strength (percentage)	6 (24)	18 (64.3)	*P* <0.05
Diabetes mellitus (%)	10 (40)	18 (64.2)	*P* = 0.08
Hypertension (%)	14 (56.0)	25 (89.2)	*P* < 0.05
Coronary artery disease (%)	1 (4)	4 (14.2)	*P* = 0.213

**Table 2:** Patient clinical parameters.

	**Control**	**ESRD**	***P* value**
Blood urea nitrogen (mg/dL)	32.76 ± 26.21	64.64 ± 19.05	*P* < 0.05
Creatinine (mg/dL)	1.99 ± 1.92	9.67 ± 3.41	*P* < 0.05
Estimated glomerular filtration rate (ml/min)	63.47 ± 36.42	8.20 ± 13/57	*P* < 0.05
Indoxyl sulfate (μM)	23.19 ± 33.28	222.81 ± 90.67	*P* < 0.05
Irisin (pg/mL)	99.77 ± 93.29	64.62 ± 32.64	*P* < 0.05
Myostatin (ng/mL)	2.17 ± 3.66	1.03 ± 0.79	*P* = 0.073
Interleukin 6 (pg/mL)	3.69 ± 6.91	10.73 ± 22.60	*P* = 0.07

### Skeletal muscle fibrosis developed in CKD mice via TGF beta signaling

**Figure 2:**
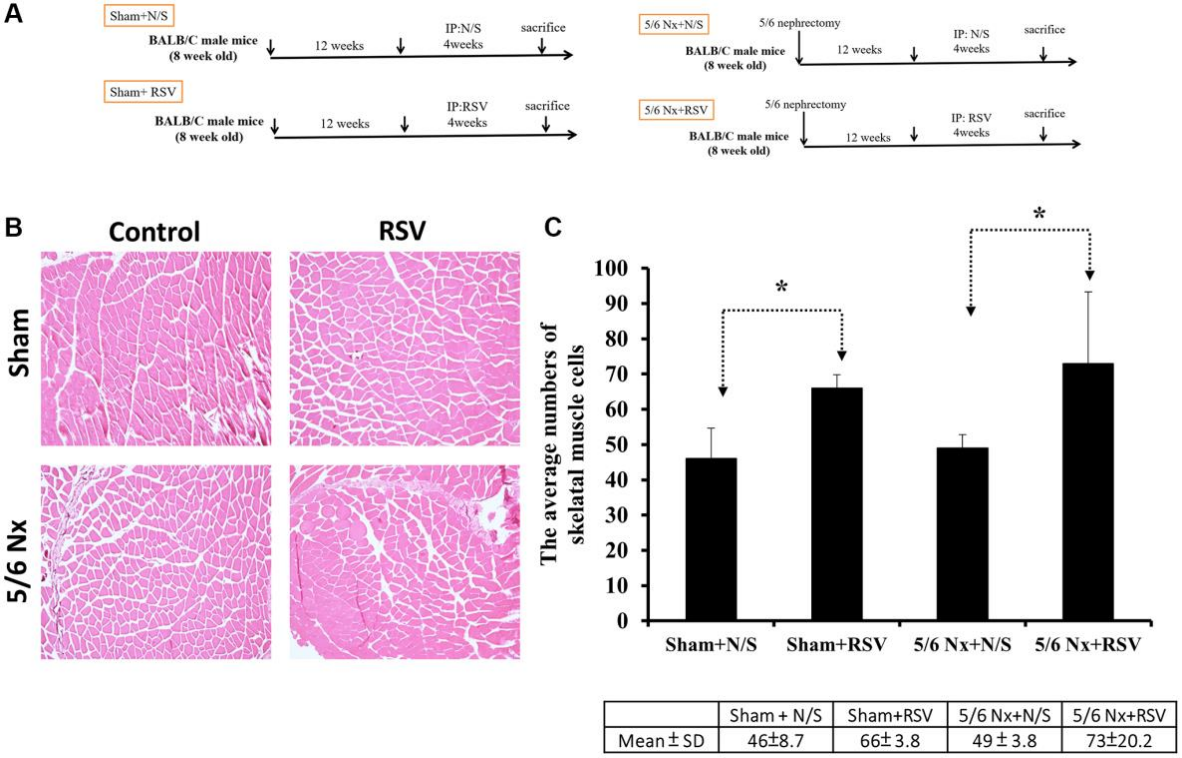
**Pathologic change in skeletal muscle in mice with 5/6 nephrectomy.** (**A**) Experimental design. (**B**) H&E staining of skeletal muscle tissues. (**C**) Number of nuclei within the skeletal muscle for each group. ^*^p < 0.05.

**Figure 3:**
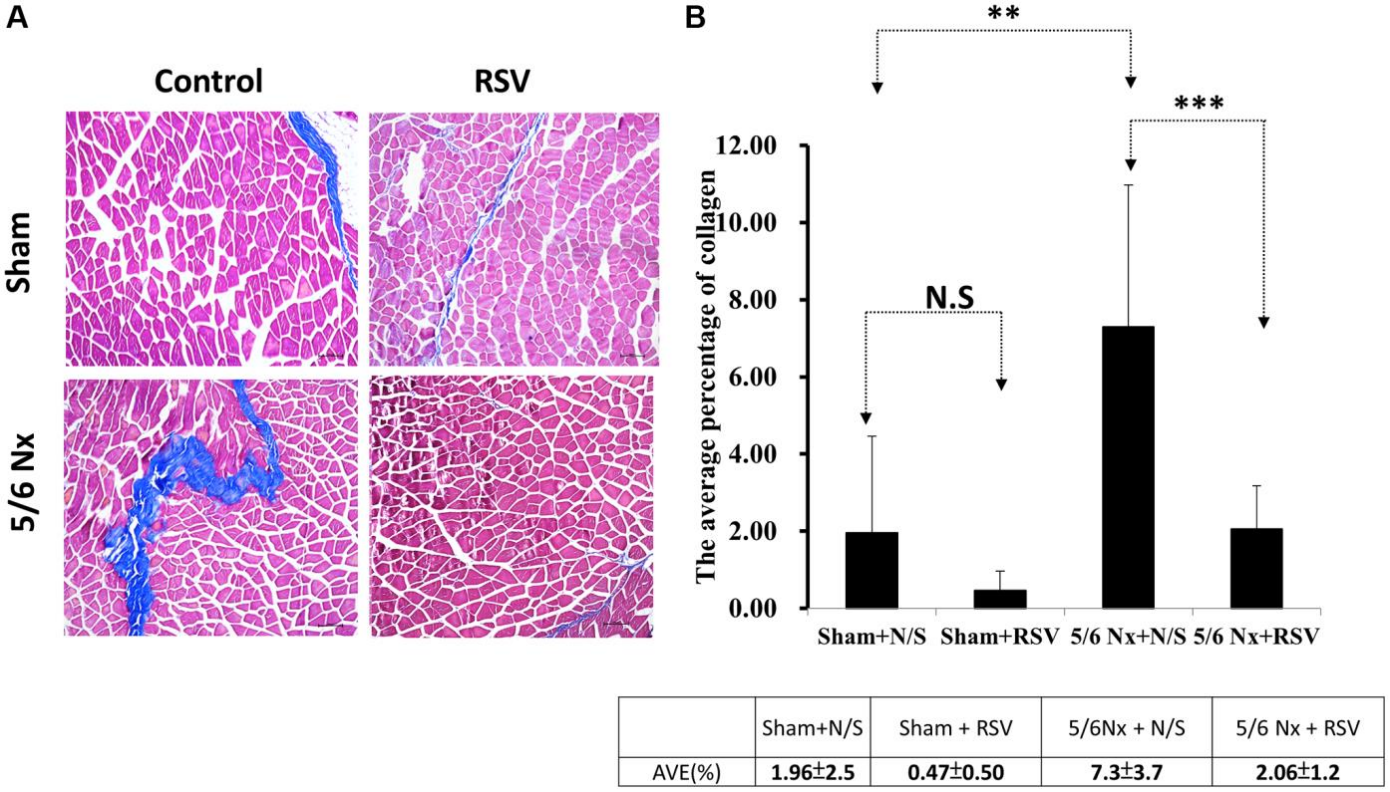
**Area of collagen in skeletal muscle tissue in mice with 5/6 nephrectomy.** (**A**) Masson’s trichrome staining of collagen fibers. (**B**) Area of collagen within the skeletal muscle was quantified for each group. ^**^Sham + N/S vs. 5/6Nx + N/S, *p* < 0.05; ^***^5/6Nx + N/S vs. 5/6 Nx + RSV, *p* < 0.05.

**Figure 4:**
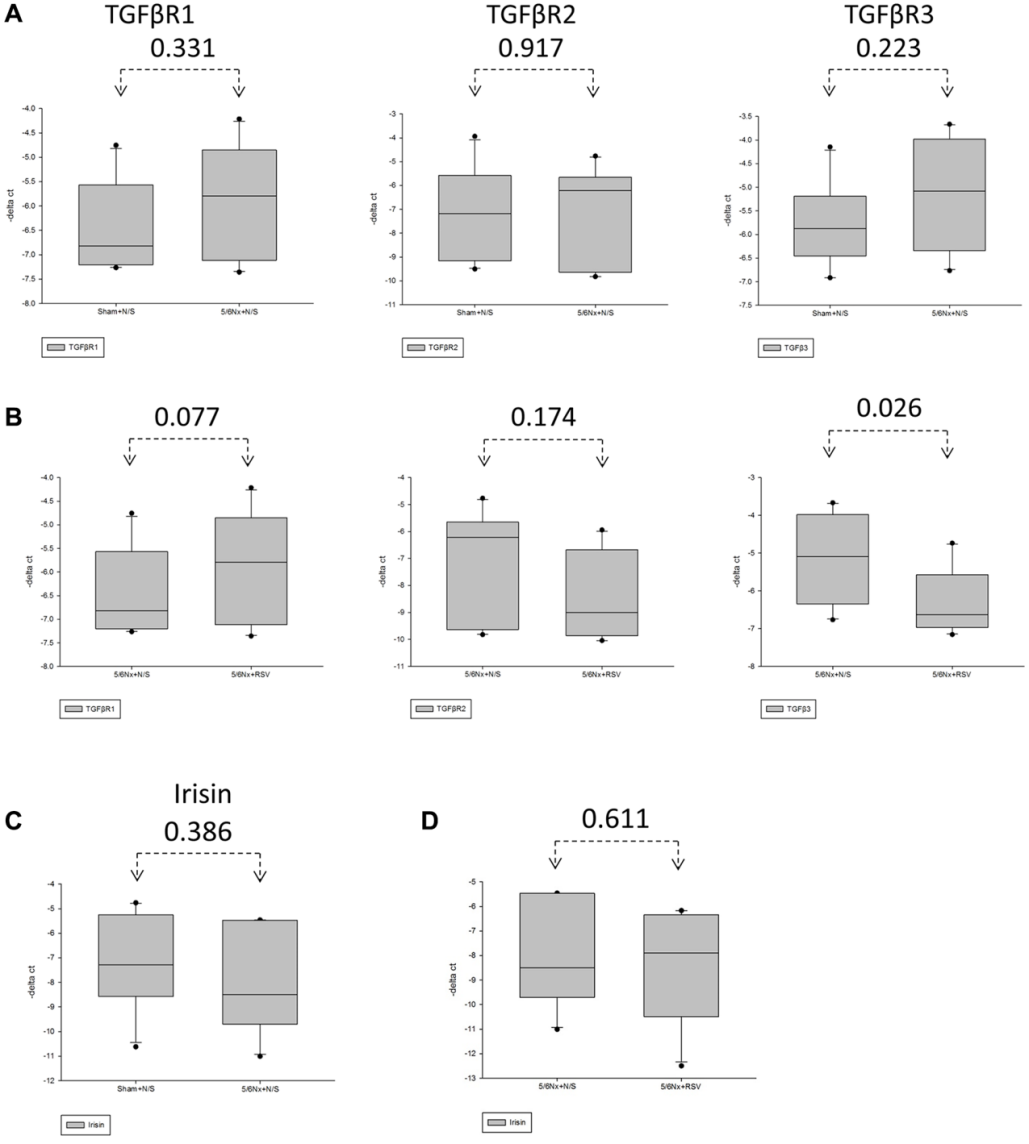
**Expression of TGF-β receptor and irisin was downregulated in mice with 5/6 nephrectomy.** (**A**, **C**) The expression of TGF-βR1, TGF-βR2 and TGF-βR3 in the gastrocnemius in between the sham group and the 5/6 nephrectomized mice through real-time PCR. (**B**) Expression of TGF-βR1, TGF-βR2 and TGF-βR3 in 5/6 nephrectomized mice with and without resveratrol (RSV) treatment through real-time PCR. (**C**) Expression of irisin in sham group and the 5/6 nephrectomized mice through real-time PCR. (**D**) Expression of irisin in the 5/6 nephrectomized mice with and without RSV treatment through real-time PCR.

**Figure 5:**
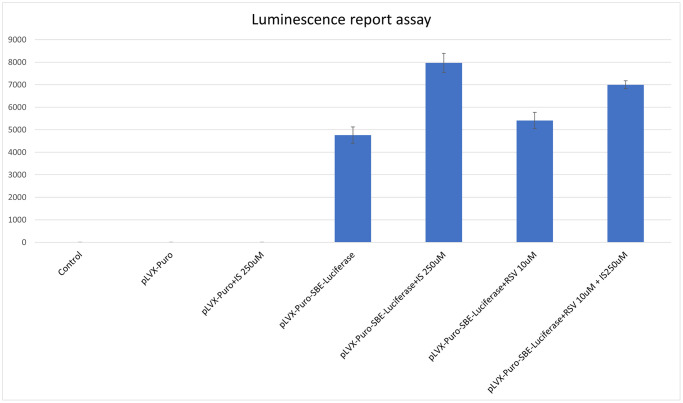
**Expression of Smad increased in C2C12 cells treated with indoxyl sulfate.** SMAD Luciferase reporter assays demonstrated the indoxyl sulfate increased the SMAD expression at concentration of 250 uM (*p* < 0.05). The treatment of resveratrol (10 uM) decreased the expression of Smad in C2C12 cells co-treated with indoxyl sulfate. ^*^p < 0.05, when comparing to the C2C12 cells treated with lentiviral reporting assay. Abbreviations: IS: indoxyl sulfate; pLVX-Puro: plasmid lentiviral expression vector; pLVX-Puro-SBE-Luciferase: plasmid lentiviral expression vector with Smad binding element luciferase; RSV: resveratrol.

**Figure 6:**
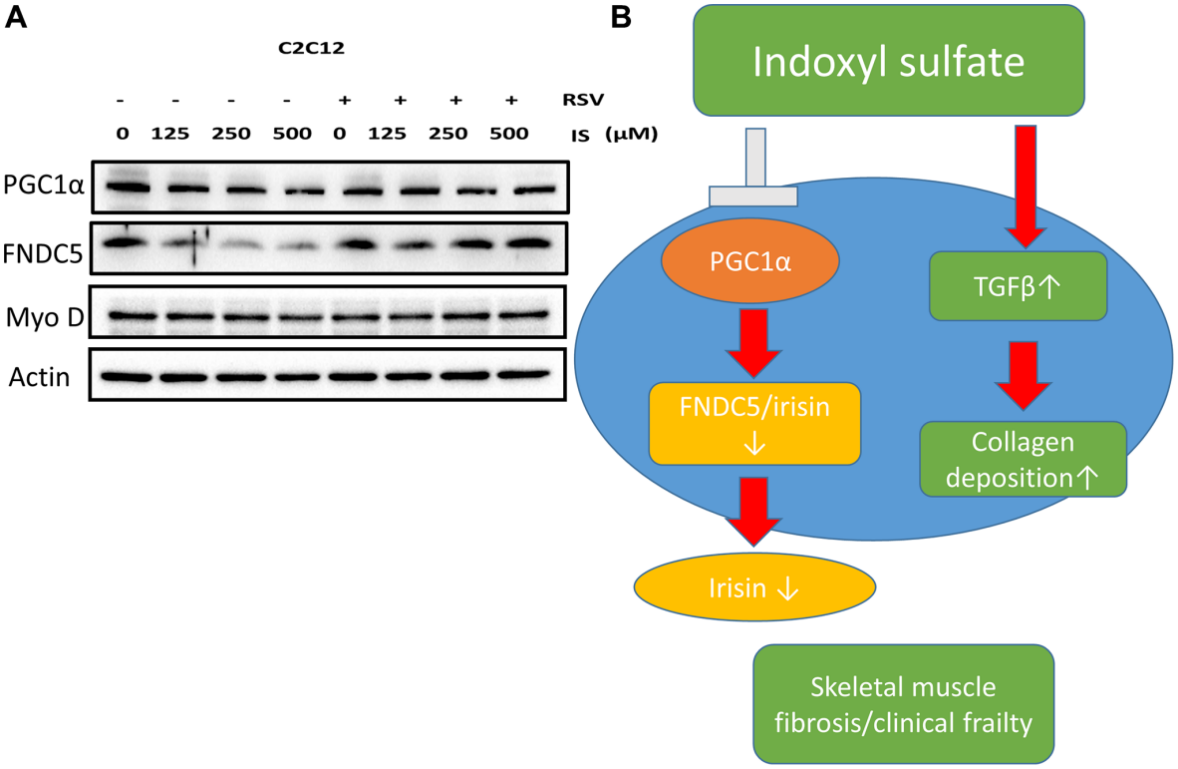
**Treatment with RSV ameliorated IS-induced PGC-1α–FNDC5 dysfunction.** (**A**) Protein levels of PGC-1α, FNDC5, myoD, and actin were examined using Western blotting. (**B**) Graphic abstract.

To investigate the association between sarcopenia and CKD, we compared the extent of pathologic change of skeletal muscle in mice with and without 5/6 nephrectomy. The experimental protocol is shown in Figure 2A. The number of skeletal muscle cells was similar in the control group and in the treatment group (46 ± 8.70 and 49 ± 3.83, respectively; Figure 2B, 2C). Treatment with RSV increased the number of skeletal muscle cells in the control groups (46 ± 8.7 vs. 66 ± 3.8, *p* < 0.05) and in the treatment groups (49 ± 3.8 vs. 73 ± 20.2, *p* < 0.05; Figure 2B, 2C). The area of collagen was higher in the treatment group than in the control group (7.3% ± 3.67% vs. 1.96% ± 2.5%, *p* < 0.01; Figure 3A, 3B). Treatment with RSV significantly reduced the area of collagen in the treatment group (7.3% ± 3.7% in the mice that underwent nephrectomy and 2.06% ± 1.2% in the mice that underwent nephrectomy and received RSV, *p* < 0.001; Figure 3A, 3B). Furthermore, the expression of TGF-β1 (*p* = 0.331), TGF-β2 (*p* = 0.917), and TGF-β3 (*p* = 0.223) increased more in the treatment group than in the control group. Treatment with RSV suppressed the expression of TGF-β1 (*p* = 0.077), TGF-β2 (*p* = 0.174), and TGF-β3 (*p* = 0.025) in the control group (Figure 4A, 4B). Figure 4C illustrated the expression of irisin between sham group and 5/6 nephrectomized mice. The expression of irisin was similar. The Figure 4D demonstrated the irisin expression between 5/6 nephrecomized mice with and without resveratrol treatment. The irisin expression was similar. Figure 5 illustrated the activity of Smad by luciferase reporting assay by C2C12 cells. For C2C12 treated with indoxyl sulfate 250 uM, the expression of Smad increased (*p* < 0.05). The resveratrol treatment decreased the expression of Smad in C2C12 cells with indoxyl sulfate treatment (Figure 5). These findings indicate that accumulation of collagen fiber was higher in the treatment group than in the control group. Treatment with RSV reduced the accumulation of collagen fiber by regulating TGF-β expression. According to clinical samples, irisin expression was significantly lower among patients with ESRD than among those without ESRD. We examined the expression of irisin in mice. As shown in Figure 4C, expression of irisin was lower in the treatment group than in the control group. This implies an association of irisin with CKD-induced sarcopenia. To explore the mechanism of irisin expression in skeletal muscle cells in patients with CKD, we performed additional *in vitro* experiments. We treated C2CL12 cells with different doses (0, 125, and 500 μM) of IS for 24 h. To reverse the toxic effect of IS, RSV was added to the IS-treated cells for 24 h. For cells treated with IS for 24 h, the levels of PGC-1α, FNDC5, and myoD decreased in a dose–dependent manner (Figure 6A). Treatment with RSV reversed the IS-mediated decrease in the levels of PGC-1α, FNDC5, and myoD. The Figure 6B illustrated graphic abstract of the study. Our data indicates that dysfunction of the PGC-1α, FNDC5, and TGF-β collagen axis might result in sarcopenia in patients with CKD. Treatment with RSV may repair CKD-induced damage by repairing the PGC-1α, FNDC5, and TGF-β collagen axis.

## DISCUSSION

We illustrated that IS is associated with sarcopenia in patients with advanced CKD. The protective effect of irisin is counteracted by IS. In mice who underwent 5/6 nephrectomy, deposition of collagen, the development of TGF-β in skeletal muscle tissues, and expression of the TGF-β and TGF-β3 receptors increased. In the *in vitro* study, treatment with IS reduced the expression of FNDC5 in a dose–dependent manner at 24 h. Treatment with RSV reversed the IS-mediated decrease in the expression of FNDC5 at 24 h.

From the clinical study, IS was associated with lower handgrip strength in patients with advanced CKD. In 5/6 nephrectomized mice, the percentage of collagen was higher than in the mice in the control group. This demonstrates that the factors associated with fibrosis within skeletal muscle are activated in the uremic milieu. The activation of TGF-β is demonstrated in our *in vivo* study. The transcriptional level of TGF-β3 and TGF-β receptors increased in 5/6 nephrectomized mice. TGF-β has been regarded as the profibrotic hormone, and its release from the extracellular matrix could be induced by a specific stimulating factor such as oxidative stress or insulin resistance commonly noticed in the uremic milieu [18–21]. The activity of PPARγ could downregulate TGF-β via direct inhibition or the release of microRNA (miRNA) associated with fibrosis, such as miR-133-5p [22, 23]. The *in vivo* study demonstrated the similar number of nuclei in the skeletal muscle, and the myoD expression was similar after IS treatment in the *in vitro* study. The clinical data demonstrated that the skeletal muscle mass did not have the association with the concentration of indoxyl sulfate [24]. Therefore, the IS might not influence the skeletal muscle viability or the direct destruction on the myotube. The disturbance of the metabolism might be the more possible influence. IS serves as the source of oxidative species in the uremic milieu. After entering the cells, IS could directly induce oxidative stress or influence the TCA cycle of the mitochondria [25]. IS could activate the production of TGF-β via the expression of atrogin-1 and myostatin [26]. Consequently, decreasing the IS burden is essential for preserving the skeletal muscle function.

RSV mitigates the effects of IS. First, RSV could serve as a scavenger of reactive oxygen species by reducing inflammation and profibrotic factors [27, 28]. Second, RSV could reduce the activity of hepatic sulfotransferase, which is essential for converting tryptophan into IS [29]. Treatment with RSV reduced IS concentrations in our *in vivo* experiments [30]. In our *in vivo* study, treatment with RSV reduced the formation of collagen within the skeletal muscle in 5/6 nephrectomized mice. Our *in vitro* study also demonstrated that RSV increased the production of PGC-1α at 24 h with IS concentrations of 125 and 500 μM. In a previous study, RSV maintained mitochondrial function by activating PGC-1α and SIRT-1, which reduced PGC-1α acetylation [31]. The activation of PGC-1α increased insulin sensitivity in skeletal muscle and brown adipose tissues. However, in another study, RSV only increased PGC-1α activity in C2C12 mitochondria under specific conditions, such as PGC-1α overexpression or SIRT-1 knockdown [32]. Our study is the first to demonstrate causality. Additional research on the underlying mechanisms, especially the interactions between IS, RSV, PGC-1α, FNDC5, and sirtuin are warranted.

The release of irisin from skeletal muscle is mainly regulated by exercise. Contraction of skeletal muscle stimulates expression of FNDC5, which is cleaved into irisin. Expression of FNDC5 is modulated by the upstream cofactors PPARγ and PGC-1α [33], which facilitates thermogenesis via upregulation of UCP-1 in the mitochondria within white adipose tissue [34]. Beyond thermogenesis, irisin plays an important role in osteoblast formation and alleviates ventricular hypertrophy via activation of the PI3K/Akt/AMPK pathway [35, 36]. From the perspective of alleviating sarcopenia and restoring handgrip strength, irisin could potentially address the underlying causes of systemic dysfunction. Furthermore, the decrease in skeletal muscle mass might not be sufficient to stimulate the release of irisin. Results from our *in vitro* experiment indicate that the expression of PGC-1α and FNDC5 decreased significantly in skeletal muscle. A possible reason is that IS caused oxidative stress, which stimulated the expression of PGC-1α to compensative. Expression of PGC-1α is influenced by the severity of oxidative stress. Reactive oxygen species increase the ratio of cytosol NAD^+^/NADH and activate SIRT-1 [37]. Increased SIRT-1 expression further modulates mitochondrial biogenesis to confront the oxidative stress by increasing SIRT-3 expression or modulating the MnSOD2 [38]. Therefore, expression of FNDC5 at 125 and 250 μM at 24 h enhanced glucose uptake in skeletal muscle in a self-regulating manner under conditions of oxidative stress [36]. Consequently, the protective effect of irisin was diminished. Augmenting endogenous irisin production may be useful for treating uremic sarcopenia in patients with CKD.

This study has several limitations. First, we demonstrated the association between plasma irisin and IS in patients with ESRD and sarcopenia. However, neither pathologic nor radiologic evidence was collected. Therefore, results from the clinical study might not correlate with those of the *in vivo* study. Second, we did not measure upstream factors for the expression of irisin in skeletal muscle. Third, factors regulating the expression of PGC-1α, such as SIRT-1, were not examined. Fourth, several factors associated with epigenetic modifications, such as NO66, were not examined. Neither epigenetic modifications nor transcription factors under conditions of stress were examined. Besides, the resveratrol increased the cell number in the mice receiving sham operation. A possible reason was that the resveratrol increased the endogenous manganese superoxide dismutase and reduced the reactive oxidative species thereafter. We sacrificed the mice at the relatively young age [39]. Therefore, the supplement of RSV might enhance the cell number. A longer housing duration might be essential to evaluate the effect of IS for aging process. Fifth, we did not compare the efficacy of IS-lowering agent with resveratrol. The measurement of IS in 5/6 nephrecomized mice was not performed although IS concentration increased in 5/6 nephrecomized mice in other literatures [40]. Further experiments with AST-120 might be essential to assess the efficacy in alleviating the fibrosis in skeletal muscle. Finally, to assessing the frailty in C57/b6 should provide more translational explanation for the frailty, such as the inverted cling grip strength in rotarod [41, 42]. To assess the frailty, the longer housing duration, such as 12–18 months, is essential for C57/b6 mice [42]. Further functional assessment could provide more evidences in the mechanism of IS and the frailty.

In conclusion, IS is associated with sarcopenia in patients with ESRD. IS may downregulate the expression of the PGC-1α–FNDC5 axis in patients with CKD. Furthermore, treatment with RSV ameliorates CKD-induced damage by repairing the PGC-1α, irisin, and TGF-β collagen axis.
